# Mammographically detected spicules associated with malignant breast tumors frequently harbor additional tumor foci

**DOI:** 10.2478/raon-2025-0041

**Published:** 2025-06-21

**Authors:** Heli Tuomainen, Mazen Sudah, Sarianna Joukainen, Vesa Kärjä, Amro Masarwah, Otto Jokelainen, Hidemi Okuma

**Affiliations:** 1 Department of Clinical Radiology, Diagnostic Imaging Center, Kuopio University Hospital, Kuopio, Finland; 2 Clinical Radiology, Institute of Clinical Medicine, School of Medicine, University of Eastern Finland, Kuopio, Finland; 3 Department of Plastic Surgery, Division of Surgery, Kuopio University Hospital, Kuopio, Finland; 4 Department of Clinical Pathology, Diagnostic Imaging Center, Kuopio University Hospital, Kuopio, Finland; 5 Pathology and Forensic Medicine, Institute of Clinical Medicine, School of Medicine, University of Eastern Finland, Kuopio, Finland

**Keywords:** spicule, breast cancer, margin assessment, breast conserving surgery

## Abstract

**Background:**

On imaging, malignant breast masses are commonly associated with spicules. To the best of our knowledge, the clinical significance of such spiculae has not been previously studied, and no surgical guidelines are available for the management of mammographically detected spiculations.

**Patients and methods:**

Between April 2018 and December 2019, all consecutive breast-conserving surgery -patients with invasive malignant lesions, who required intraoperative radiological breast specimen assessment with tomosynthesis, were retrospectively included in this analysis. The tumors were classified into two groups: those with spiculated margins as the dominant feature, and those with other distinct morphological characteristics. Spicule visualization, length, and distribution were evaluated in both groups using pre- and intraoperative imaging and compared with the histopathological features of the spicules.

**Results:**

In total, 162 invasive lesions were evaluated. The presence of spicule-associated additional tumor foci was a common finding; 67.6% of the spiculated tumors and 48.9% of the other tumors had additional foci. Most additional tumor foci were within 1 cm of the tumor edge. The mean pathologically measured distance from the main tumor margin to the spicule-associated additional tumor foci was 4.3 ± 2.8 mm. Compared to the maximum spicule length determined from intraoperative images (9.5 ± 5.1 mm), the distance of actual tumor infiltration was much shorter, and a very weak correlation was observed.

**Conclusions:**

Breast tumor spicules harbor additional tumor foci, which may lead to margin positivity and potential reoperation. Additional research is necessary to determine the actual tumor burden and clinical significance of spicules.

## Introduction

Breast cancer is a heterogeneous disease that can be multifocal in nature, irrespective of tumor size or histology, which poses challenges in its management. Early results on mastectomy specimens showed that only 37% of T1–2 invasive breast cancers were unifocal, while the rest had additional tumor foci.^1^ Such additional foci can lead to positive resection margins after breast-conserving surgery (BCS), and thus, an increased risk of reoperation or local recurrence. This highlights the importance of thorough preoperative assessment to identify any additional tumor foci that might not be apparent upon initial examination. Based on our own observations, most occult tumor foci detected after BCS are localized within tumors’ spicules.

A spiculated breast lesion can be defined as a mass or architectural distortion with thin lines radiating from its margins.^2^ Spicules are common in malignant breast lesions yet can be observed in both benign and malignant breast processes.^2^ The exact mechanisms underlying malignant spicule formation beyond the tumor mass are not fully understood, and its clinical significance is unknown. It is speculated that spiculation involves two time-dependent steps: cancer cell invasion initially associated with a desmoplastic stromal reaction, followed by adipose tissue invasion and/or involvement of Cooper’s ligaments by carcinoma tissue.^3^ Adipose tissue can release hormones and active adipokines that participate in the regulation of the immune response, cancer metabolism, and energy balance.^4,5^ Adipocyte–cancer cell crosstalk is important for the desmoplastic reaction, which might contribute to spiculation.^6^ Furthermore, the extracellular matrix is not a passive bystander in cancer progression.^7^ Collagen fibers create pathways that cancer cells can use to invade the environment.^8^ Consequently, spicules are not only connective tissues but may also contain tumor cells.^8^ Since focal invasion begins at the base of the spicule, it has been suggested that tumor cells should be more abundant at the spicule’s base than at its end.^8^

Spiculations pose also a challenge for imaging. Mammographic features cannot reliably differentiate whether the spiculated lesion is benign or malignant, resulting in the need for a biopsy unless the lesion is indisputably postsurgical fibrotic tissue.^9^ Spiculations are difficult to detect in dense breast.^3^ Although spicules are usually visible on full-field digital mammography (FFDM), summations from normal fibroglandular tissue might hinder the evaluation of spicules on conventional mammograms.^8^ Digital breast tomosynthesis is a three-dimensional imaging modality that eliminates tissue superimposition and provides a clearer view of the lesion and its margins and spicules as well as associated architectural distortions, making it easier to differentiate spicules from the summations of the fibroglandular tissue.^8^ Furthermore, tomosynthesis has recently been shown to be superior to other mammographic imaging modalities for the evaluation of resected specimens.^10^

To the best of our knowledge, no studies have been conducted to assess (and no guidelines are available on) the surgical removal of mammographically detected spicules or the frequency of occurrence of tumor foci in spicules. Therefore, this study was designed to evaluate the frequency and nature of additional occult malignant lesions and their relationship with spicule size and quantity, as visualized by specimen tomosynthesis.

## Patients and methods

### Study population

The study population comprised consecutive BCS patients with invasive malignant lesions who required radiological intraoperative breast specimen assessment with tomosynthesis at our tertiary university hospital between April 2018 and December 2019. Patients who received neoadjuvant chemotherapy were excluded from this study. The Chair of the Hospital District waived the need to obtain written informed consent from patients owing to the retrospective nature of the analysis. Some patients were included in previously published studies, in which the accuracy of different specimen radiography techniques was evaluated.^10,11^ All investigations were conducted by experienced breast radiologists and surgeons according to the relevant national guidelines and principles of the Declaration of Helsinki.

All patients were evaluated preoperatively with a minimum of two-view mammography and bilateral breast ultrasound (US). Suspicious lesions were evaluated using additional lateral and spotcompression views and/or tomosynthesis as needed. All mammograms were re-evaluated upon referral by a specialized breast radiologist, and further workup was performed if deemed necessary. The patients underwent US- or stereotactic-guided core or vacuum-assisted biopsies and were histologically diagnosed with invasive breast cancer. If lesions were found in both breasts, each specimen was evaluated separately.

### Lesion localization and surgery

Non-palpable tumors were localized preoperatively using at least one guidewire and excised en bloc from the subcutaneous area to the muscle. The overlying skin was removed in patients with superficial lesions. Intraoperatively, the specimens were placed on a Styrofoam slab, fixed with wooden sticks and the location of the excision was anatomically marked. Metallic clips were placed directly on the specimens to indicate their orientation. The fascia posterior to the tumor was removed and fixed aside if removed separately. The specimen was then placed in a plastic container and immediately transported to the Breast Radiology Unit, where the margins were evaluated intraoperatively to ensure the removal of both the tumor and spicules to achieve a healthy lateral macroscopic surgical margin of ≥ 1 cm from the tumor’s edge, in accordance with national guidelines, and hence to achieve microscopically negative margins (defined as no “tumor-on-ink”).

### Histopathological evaluation

The specimens were measured and photographed, and ink was applied to their margins. Subsequently, the specimens were sliced either in the frontal or sagittal planes at 5 mm intervals, depending on the size of the specimen. Histopathological data, including the margin status, tumor size, histological grade, estrogen receptor (ER) status, progesterone receptor (PR) status, human epidermal growth factor receptor 2 (HER-2) status (using both immunohistochemistry and chromogenic in situ -hybridization), and the Ki-67 proliferation index, were evaluated and recorded in a structured manner. For this study, two specialized pathologists (OJ and VK) retrospectively reviewed the surgical specimens for spicule histology. The presence of spicules and whether they contained in situ and/or invasive carcinoma was evaluated. Furthermore, the distance between these additional lesions and the index tumor’s margin was measured. The cancers were classified into four subtypes according to the St. Gallen consensus panel.^12^

### Imaging protocol and analysis

Patients were referred to our hospital for consultation and treatment from two screening units, two district hospitals and multiple primary healthcare centers representing a catchment area of approximately 250,000 people. In general, basic breast examinations were performed in accordance with national guidelines, that include a minimum of two-view FFDM, a lateral view on the affected side combined with spot compression orthogonal views or DBT, depending on the availability of equipment, followed by bilateral whole-breast and axillary ultrasound examinations. Intraoperatively, each specimen was imaged bare, without tissue compression by 2D FFDM in craniocaudal and lateral projections (Selenia Dimensions® breast tomosynthesis system, Hologic Inc., Bedford) followed immediately by tomosynthesis (images reconstructed into a series of 1-mm-thick slices at 1-mm intervals). One of the two experienced breast radiologists (AM or MS) retrospectively and blindly evaluated each preoperative mammogram and recorded the dominant radiological features of the lesions using the fifth edition of the Breast Imaging Reporting and Data System (BI-RADS) lexicon.^13^ Additionally, the preoperative two-view mammograms and intraoperative specimen tomosynthesis images were evaluated by one of the two experienced breast radiologists (HO or MS) who were blinded to the clinical and pathological features of the lesions. To minimize possible bias, the observers analyzed the pre- and intraoperative images separately, with a minimum interval of two weeks in between. The presence, distribution, quantity, maximum length, and thickness of spicules were evaluated. According to their margins, tumors were divided into spiculated and non-spiculated groups. A spiculated tumor was defined as a mass with lines radiating from its margins as a dominant morphological feature. A tumor with morphologically circumscribed, microlobulated, angular or indistinct margin was referred to as “non-spiculated tumor”, even though it may exhibit a few spicules along certain sections of its edge.

### Statistical analysis

All statistical analyses were performed using SPSS for Windows version 29 (IBM Corporation, Armonk, NY, USA). Statistical significance was set at P < 0.05. Pearson’s correlation coefficient or McNemar’s test was used to assess the association between the features of spicules on pre- and intraoperative images as well as the relationship between the measurements by the imaging modalities and histopathology. Cohen’s Kappa was used to assess the agreement between the distribution of the pathologically and radiologically categorized measurements. Pearson’s χ^2^ test was used to examine differences in the frequency of cancer subtypes and the presence of additional tumors in spicules between spiculated and non-spiculated masses. An unpaired t-test or analysis of variance (ANOVA) was used to assess the mean difference in the radiological and pathological measurements in relation to the presence of biomarkers such as ER, PR, HER-2, Ki-67, and histological grade, as well as the mean difference in tumor size in relation to the presence of additional tumors in spicules.

An r-value of 0.75–1 indicated a very strong correlation, an r-value of 0.50–0.75 indicated a strong correlation, an r-value of 0.25–0.50 indicated a weak correlation, and an r-value ≤ 0.25 indicated a very weak correlation.^14^

## Results

A total of 162 invasive lesions from 156 women (mean age: 63.0 ± 10.2 years, range: 33–95 years) fulfilled the inclusion criteria and were evaluated. The patient and tumor characteristics are presented in Table 1, and the mammographic characteristics of the tumors are presented in Table 2.

**TABLE 1.** Characteristics of the patients and tumors

	Number (%)
Patients	156
Lesions	162
Mean age, years (range)	63.0 ± 10.2 (33–95)
Histology	
Invasive ductal	125 (77.2)
Invasive lobular	29 (17.9)
Others^*^	8 (4.9)
Size of tumor, mm (range)	16.2 ± 10.0 (2–60)
Grade	
1	51 (31.5)
2	87 (53.7)
3	24 (14.8)
T-stage	
T1	122 (75.3)
T2	38 (23.5)
T3	2 (1.2)
N-stage	
N0	117 (72.2)
N1	39 (24.1)
N2	5 (3.1)
N3	1 (0.6)
ER status	
Positive	149 (92.0)
Negative	13 (8.0)
PR status	
Positive	144 (88.9)
Negative	18 (11.1)
HER-2 status	
Positive	11 (6.8)
Negative	151 (93.2)
Ki-67 status	
Lower (≤ 14 %)	60 (37.0)
Higher (> 14 %)	101 (62.3)
Data missing	1 (0.6)

1 The data are shown as the number (percentage) or the mean ± standard deviation (range).

Others*: = invasive micropapillary carcinoma, papillary carcinoma, mucinous cancer × 4, medullary like and cribriform carcinoma.

1 ER = estrogen receptor; PR = progesterone receptor; HER-2 = human epidermal growth factor receptor 2

**TABLE 2.** Mammographic features of the tumors and the lesion descriptions according to Breast Imaging Reporting and Data System (BI-RADS) 5^th^

Breast composition	Number (%)
A	49 (30.2)
B	84 (51.9)
C	26 (16.0)
D	3 (1.9)
Findings	
Mass	128 (79.0)
Calcification	25 (15.4)
Architectural distortion	6 (3.7)
Asymmetry	2 (1.2)
Invisible on mammography	1 (0.6)
Mass shape	
Oval	14 (10.9)
Round	61 (47.7)
Irregular	53 (41.4)
Mass margin	
Circumscribed	3 (2.3)
Obscured	3 (2.3)
Microlobulated	31 (24.2)
Indistinct	23 (18.0)
Spiculated	68 (53.1)

1 Data are shown as the number (percentage).

A summary of the features of the spicules observed in the pre- and intraoperative images is shown in Table 3. There was no statistically significant difference in the presence or distribution of spicules between the pre- and intraoperative images. However, there was a statistically significant difference and a strong positive correlation between the pre- and intraoperative images was found regarding the number and maximum length of the spicules.

**TABLE 3.** Presence and distribution of spicules in the pre- and intraoperative images

	Pre (%)	Intra (%)	P value (r^*^)
Number of spicules	12.2 ± 6.3	13.0 ± 6.2	< 0.001 (0.577)
Maximum length of spicules (mm)	7.3 ± 5.0	9.5 ± 5.1	< 0.001 (0.564)
Presence of spicules			0.648
Yes	97 (59.9)	100 (61.7)	
No	65 (40.1)	62 (38.3)	
Radial distribution of spicules			0.192
< 25 %	8 (8.2)	7 (7.0)	
25–50 %	23 (23.7)	22 (22.0)	
50–75 %	21 (21.6)	18 (18.0)	
75–100 %	45 (46.4)	53 (53.0)	

* = Pearson’s coefficient; Intra = intra-operative images; Pre = pre-operative images

1 The data are shown as the mean ± standard deviation or the number (percentage).

The mean pathologically measured distance from the main tumor margin to the additional spicule-associated tumor was 4.3 ± 2.8 mm. Compared to the maximum spicule length measured in the intraoperative images (9.5 ± 5.1 mm), the distance of actual tumor infiltration was much shorter, and the correlation was very weak (P = 0.055, r = 0.234).

**Figure 1.**
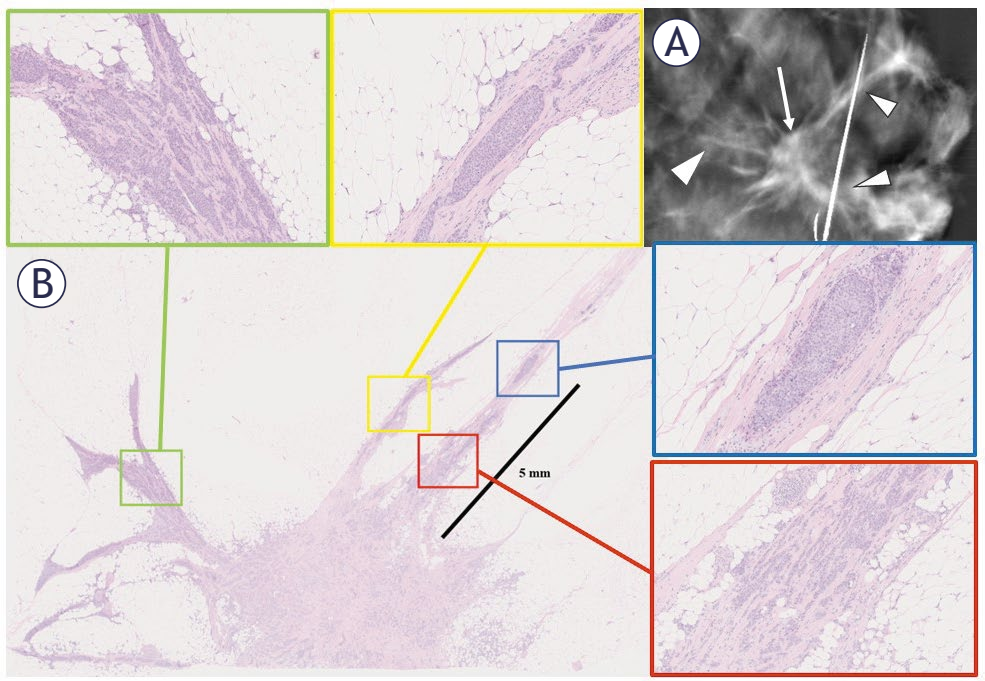
A 14 mm spiculated invasive ductal carcinoma (IDC) excised en bloc with spiculations. A tomosynthesis specimen radiogram at the level of the tumor documents the total removal of both the guidewire localized tumor (**A**; arrow) and the associated spicules (arrowheads). The histopathological analysis (**B**, H&E; 10 × magnification) showed that all the spicules contained additional tumors: the spicule shown in the red box contained IDC (100 × magnification); one spicule contained ductal carcinoma in situ (DCIS) (blue box, 150 × magnification); other spicules contained both DCIS and IDC in green (100 × magnification) and yellow (150 × magnification) boxes.

In terms of their distance from the main tumor margin, most tumors in the spicules were < 5 mm away (55, 60.4%), some were 5–9 mm away (30, 33.0%), and a small proportion were ≥ 10 mm away (6, 6.6%). The distribution of spicule lengths in the preoperative images was as follows: 38 (39.2%) were < 5 mm in length, 34 (35.1%) were 5–9 mm in length, and 25 (25.8%) were ≥ 10 mm in length. The pathologically and radiologically categorized measurements showed significantly different distributions (P = 0.002) and no agreement (P = 0.66, κ = 0.04). The types of tumors found in the spicules were as follows: no additional tumor (70, 43.2%), invasive only (34, 21.0%), in situ only (31, 19.1%), and both invasive and in situ (27, 16.7%). Representative images are shown in Figures 1–Figures 3.

**Figure 2.**
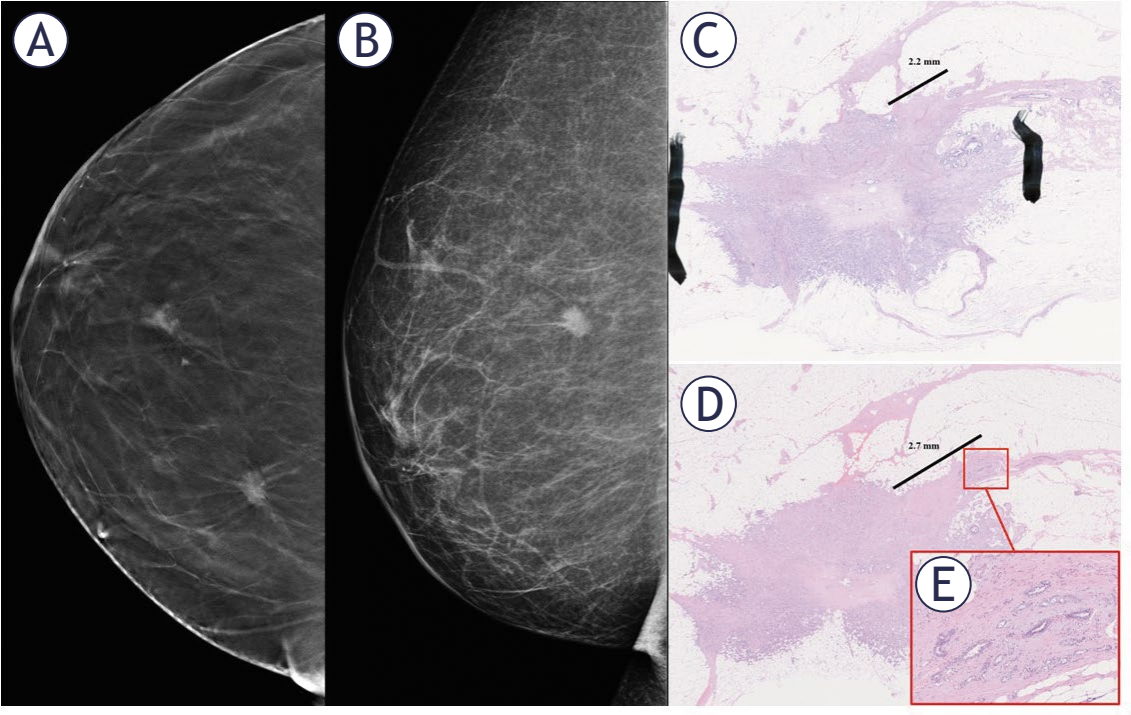
A mammogram of a nonpalpable, spiculated 12 mm invasive ductal carcinoma: a craniocaudal-tomosynthesis view **(A)** demonstrates spiculations better than full-field digital mammography in mediolateral-projection **(B)**. After breast conserving surgery, a more superficial axial section **(C)** the invasive additional cancer extends 2.2 mm from the tumor’s margin. In the deeper section **(D)** additional invasive cancer cells are visible further in the spicule extending up to 2.7 mm from the tumor’s margin highlighting that analyzing only one section of a spicule will underestimate the extent of the disease. **(E)** A magnified view of the base of the spicule.

**Figure 3.**
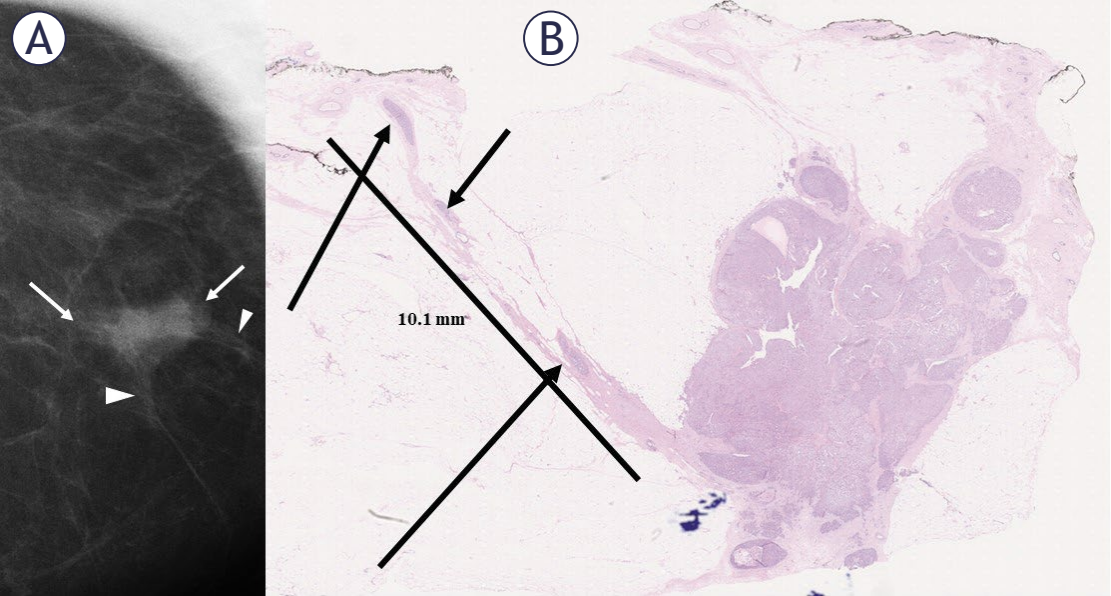
**(A)** A mediolateraloblique spot magnification view of a 13 mm mainly circumscribed, mixed papillary and invasive ductal carcinoma (arrows) with a few spicules (arrowheads). The histopathologal analysis **(B)** showed a single solid spicule with multiple foci, likely representing a continuum of low-grade ductal carcinoma in situ (arrows) extending as far as 10.1 mm from the tumor’s edge and close to the resection margin.

Tumors that had spicules containing additional tumor material were statistically larger than those that did not have spicules containing additional tumor material (17.9 ± 11.6 *v*s. 13.8 ± 6.9 mm, respectively, P = 0.006).

None of the tested biological markers (i.e. ER, PR, HER-2, Ki-67, or grade) showed any significant relationship with the distribution, number, or length of spicules, the presence of additional tumors in spicules, or their distance from the main tumor margin.

Table 4 summarizes the distribution of the tumor subtypes in relation to spiculation and the presence of additional tumors in the spicules. Luminal A and B tumors more frequently presented with spiculation as a dominant feature (χ^2^= 10.4, P = 0.016), and more additional tumors were found in spiculated masses (χ^2^ = 5.6, P = 0.018) compared to non-spiculated tumors.

**Table 4.** Distribution of tumor subtypes and presence of additional tumor material in spicules in relation to spiculated mass

	Spiculated	(%)	Non-spiculated	(%)	P value
Subtype					
Luminal A	28	41.2	32	34.0	0.016
Luminal B	39	57.4	46	48.9	
HER-2 enriched	1	1.5	8	8.5	
Basal	0	0	8	8.5	
Presence of additional tumor in spicules					
Yes	46	67.6	46	48.9	0.018
No	22	32.4	48	51.1	

1 HER-2 = human epidermal growth factor receptor 2

## Discussion

The results of this study suggest that the presence of spicule-associated tumor foci is a common finding in breast cancer, irrespective of the length or quantity of the spicules. Furthermore, in this study, the overwhelming majority of these additional foci were within 1 cm of the tumor edge. These results strongly imply that spiculation is not insignificant and should be reported and included in the preoperative surgical plan. To the best of our knowledge, there have been no previous studies on this issue, and no surgical guidelines are available for the management of mammographically detected spiculations.

Even though residual microscopic disease after surgery will most likely be eradicated by post-surgical adjuvant treatments, the presence of positive margins after resection significantly increases the risk of local relapse. Spiculated tumors have been linked to an increased risk of ipsilateral recurrence^3,15^, although low-grade tumors are more likely to exhibit a spiculated margin compared to rapidly growing high-grade tumors.^16^ In addition, during imaging, the appearance of low-grade tumors is influenced by the presence of tumor- associated fibrous connective tissue (i.e., the desmoplastic reaction).^17^ However, Moriuchi *et al*.^3^ found no statistically significant differences in the 10-year disease-free or overall survival rates between patients with spiculated and non-spiculated masses.^3^ Tabar *et al*.^18^ reported that small (1–14 mm) spiculated tumors were associated with excellent long-term survival.

While the routine removal of all spicules during surgery might decrease the risk of positive margins, it will result in larger resection, more complex surgery, potentially inferior aesthetic results, and lower patient satisfaction. Reoperation rates vary significantly among centers and surgeons, with an average rate of 20%.^19^ Our results may explain the high frequency of positive margins reported after lumpectomy.^20^ Cavity shaving has been shown to reduce the positive margin and re-excision rate by half^20^, and assumingly shaving removes a significant number of peritumoral spiculations. Finnish national guidelines recommend wide local excision of tumors, with a minimum lateral macroscopic margin of 1 cm; however, different centers have reported different reoperation rates, ranging from 8.4 to 13%.^21,22^ In our local practice, we strictly adhere to this recommendation, and based on our observations, we routinely recommend the removal of all associated spicules that, on average, are about 1 cm in length. This approach, together with our meticulous preoperative evaluation process, may partly explain our previously reported low reoperation rates of 0.5–2.1%.^10,23^

Intraoperative specimen tomosynthesis was previously shown to be superior to both FFDM and dedicated digital specimen radiography system in the evaluation of lesion size, margins, spicules and calcifications.^10^ However, in this study, intraoperative specimen tomosynthesis was not significantly superior to extensive preoperative imaging in terms of the visualization and characterization of spicules. Therefore, in our opinion, it can be used to confirm that the detected spicules have been excised. Furthermore, our results indicate that analyzing preoperative FFDM images plus additional images, including spot compression and/or tomosynthesis images, is sufficient to properly plan surgical interventions.

Other studies have reported that mammographic spiculation is a good independent prognostic factor for screening-detected invasive breast cancer.^24,25^ These favorable characteristics include ER and PR positivity as well as lower histological grade and Ki67 expression.^24,26^ Samaržija *et al*.^8^ found that longer and thinner spicules were associated with a low Ki-67 index, whereas shorter and thicker spicules were associated with a higher Ki-67 index. In our study population, we did not find any significant relationship with 1) the spicule distribution, number, or length, 2) the presence of tumors in the spicules, or 3) their distance from the main tumor margin.

The mammographically detected spicule length correlated only weakly with actual tumor infiltration, and no correlation was found with any measurable parameter that could help predict the presence or extent of tumor cell infiltration in spicules. Even though the majority of spicule-associated tumors (60.4%) were located less than 5 mm from the tumor edge, additional foci were frequently found outside this range. Therefore, even a margin of ≥ 1 cm has a 6.6% theoretical risk of positive margins.

This study has some limitations. The results should be interpreted with caution, as the spicules were not fully evaluated histologically via serial sectioning. Serial sectioning requires extensive resources in a prospective setting, whereas this study was retrospective in nature. The specimens and samples were handled and prepared according to the routine protocol of the Department of Clinical Pathology. However, this protocol does not include sampling of all spicules, some of which are not macroscopically visible. Therefore, it is likely that not all spicules were histopathologically evaluated or included in the correlation analysis with the imaging findings. Consequently, the number of tumor foci in the spicules was likely underestimated. Furthermore, the tumors examined in this study were relatively small (pT1 75.3% and pT2 23.5%), had less aggressive prognostic features, and were mostly early-stage nonpalpable cancers that required intraoperative specimen assessment. Given that the lesions that had additional tumors were statistically larger than those that did not have additional tumors, it can be assumed that larger or palpable tumors with potentially longer spicules might have more abundant additional disease. Additionally, the specimens examined in this study were obtained from a single institution.

Nevertheless, this exploratory study is the first of its kind and was conducted to verify the clinical value of spicules, which might have practical clinical potential. To reduce bias, consecutive patients were included in this study. Our results clearly highlight the need for future prospective research that includes extensive serial sectioning of the spicules.

In conclusion, the results of this study show that spicules are significant structures in that they frequently harbor tumor foci, which might result in margin positivity and potential reoperation. Additional research is necessary to determine the tumor burden and clinical significance of both spicules and their tumor foci.

## References

[1] Holland R, Veling SH, Mravunac M, Hendriks JH. (1985). Histologic multifocality of Tis, T1-2 breast carcinomas. Implications for clinical trials of breast-conserving surgery. Cancer.

[2] Franquet T, De Miguel C, Cozcolluela R, Donoso L. (1993). Spiculated lesions of the breast: mammographic-pathologic correlation. RadioGraphics.

[3] Moriuchi H, Yamaguchi J, Hayashi H, Ohtani H, Shimokawa I, Abiru H (2016). Cancer cell interaction with adipose tissue: Correlation with the finding of spiculation at mammography. Radiology.

[4] Lengyel E, Makowski L, DiGiovanni J, Kolonin MG. (2018). Cancer as a matter of fat: The crosstalk between adipose tissue and tumors. Trends Cancer.

[5] Wu Y, Li X, Li Q, Cheng C, Zheng L. (2022). Adipose tissue-to-breast cancer crosstalk: Comprehensive insights. Biochim Biophys Acta Rev Cancer.

[6] Motrescu ER, Rio MC. (2008). Cancer cells, adipocytes and matrix metalloproteinase 11: a vicious tumor progression cycle. Biol Chem.

[7] Kaushik S, Pickup MW, Weaver VM. (2016). From transformation to metastasis: deconstructing the extracellular matrix in breast cancer. Cancer Metastasis Rev.

[8] Samaržija K, Jurjević Z. (2021). Association of the imaging characteristics of desmoplasia on digital breast tomosynthesis and the Ki-67 proliferation index in invasive breast cancer. Croat Med J.

[9] Demirkazık FB, Gülsün M, Fırat P. (2003). Mammographic features of nonpalpable spiculated lesions. Clinical Imaging.

[10] Almasarweh S, Sudah M, Okuma H, Joukainen S, Kärjä V, Vanninen R (2022). Diagnostic performance of tomosynthesis, digital mammography and a dedicated digital specimen radiography system versus pathological assessment of excised breast lesions. Radiol Oncol.

[11] Almasarweh S, Sudah M, Okuma H, Joukainen S, Vanninen R, Masarwah A. (2024). Specimen tomosynthesis provides no additional value to specimen ultrasound in ultrasound-visible malignant breast lesions. Scand J Surg.

[12] Goldhirsch A, Wood WC, Coates AS, Gelber RD, Thürlimann B, Senn HJ. (2011). Strategies for subtypes--dealing with the diversity of breast cancer: highlights of the St. Gallen International Expert Consensus on the Primary Therapy of Early Breast Cancer 2011. Ann Oncol.

[13] D’Orsi CJ, Sickles EA, Mendelson EB, Morris EA, American College of Radiology (2013). ACR BI-RADS Atlas: Breast Imaging Reporting and Data System ; Mammography, Ultrasound, Magnetic Resonance Imaging, Follow-up and Outcome Monitoring, Data Dictionary.

[14] Udovicic M, Bazdaric K, Bilic-Zulle L, Petrovecki M. (2007). What we need to know when calculating the coefficient of correlation?. Biochem Med.

[15] Dalberg K, Azavedo E, Svane G, Sandelin K. (1996). Mammographic features, predictors of early ipsilateral breast tumour recurrences?. Eur J Surg Oncol.

[16] Rong XC, Kang YH, Shi GF, Ren JL, Liu YH, Li ZG (2023). The use of mammography-based radiomics nomograms for the preoperative prediction of the histological grade of invasive ductal carcinoma. J Cancer Res Clin Oncol.

[17] Lamb PM, Perry NM, Vinnicombe SJ, Wells CA. (2000). Correlation between ultrasound characteristics, mammographic findings and histological grade in patients with invasive ductal carcinoma of the breast. Clin Radiol.

[18] Tabar L, Tony Chen HH, Amy Yen MF, Tot T, Tung TH, Chen LS (2004). Mammographic tumor features can predict long-term outcomes reliably in women with 1-14-mm invasive breast carcinoma. Cancer.

[19] Landercasper J, Borgert AJ, Fayanju OM, Cody H, Feldman S, Greenberg C (2019). Factors associated with reoperation in breast-conserving surgery for cancer: A prospective study of American Society of Breast Surgeon members. Ann Surg Oncol.

[20] McEvoy MP, Landercasper J, Naik HR, Feldman S. (2018). Update of the American Society of Breast Surgeons Toolbox to address the lumpectomy reoperation epidemic. Gland Surg.

[21] Niinikoski L, Leidenius MHK, Vaara P, Voynov A, Heikkilä P, Mattson J (2019). Resection margins and local recurrences in breast cancer: Comparison between conventional and oncoplastic breast conserving surgery. Eur J Surg Oncol.

[22] Lepomäki M, Karhunen-Enckell U, Tuominen J, Kronqvist P, Oksala N, Murtola T (2022). Tumor margins that lead to reoperation in breast cancer: A retrospective register study of 4,489 patients. J Surg Oncol.

[23] Joukainen S, Laaksonen E, Vanninen R, Kaarela O, Sudah M. (2022). Dual-layer rotation: A versatile therapeutic mammoplasty technique. Ann Surg Oncol.

[24] Sturesdotter L, Sandsveden M, Johnson K, Larsson AM, Zackrisson S, Sartor H. (2020). Mammographic tumour appearance is related to clinicopathological factors and surrogate molecular breast cancer subtype. Sci Rep.

[25] Evans AJ, Pinder SE, James JJ, Ellis IO, Cornford E. (2006). Is Mammographic spiculation an independent, good prognostic factor in screening-detected invasive breast cancer?. Am J Roentgenol.

[26] Ciatto S, Morrone D, Catarzi S, Bonardi R. (1992). Breast cancer: reliability of mammographic appearance as a predictor of hormone receptor status. Radiology.

